# Smart Water Meter Using Electrical Resistance Tomography †

**DOI:** 10.3390/s19143043

**Published:** 2019-07-10

**Authors:** Chenning Wu, Martin Hutton, Manuchehr Soleimani

**Affiliations:** 1 Engineering Tomography Laboratory (ETL), Department of Electronic and Electrical Engineering, University of Bath, Bath BA2 7AY, UK; 2 Ashridge Engineering Ltd, Okehampton EX20 1BQ, UK

**Keywords:** electrical resistance tomography, smart water meter, wastewater management

## Abstract

Smart flow monitoring is critical for sewer system management. Obstructions and restrictions to flow in discharge pipes are common and costly. We propose the use of electrical resistance tomography modality for the task of smart wastewater metering. This paper presents the electronics hardware design and bespoke signal processing to create an embedded sensor for measuring flow rates and flow properties, such as constituent materials in sewage or grey water discharge pipes of diameters larger than 250 mm. The dedicated analogue signal conditioning module, zero-cross switching scheme, and real-time operating system enable the system to perform low-cost serial measurements while still providing the capability of real-time capturing. The system performance was evaluated via both stationary and dynamic experiments. A data acquisition speed of 14 frames per second (fps) was achieved with an overall signal to noise ratio of at least 59.54 dB. The smallest sample size reported was 0.04% of the domain size in stationary tests, illustrating good resolution. Movements have been successfully captured in dynamic tests, with a clear definition being achieved of objects in each reconstructed image, as well as a fine overall visualization of movement.

## 1. Introduction

Process tomography provides measurements of concentration distribution and flow profile within a process instrument by collecting data via remote sensors. Electrical tomography is one of the most extensive tomographic modalities that can provide cross-sectional profiles of the distribution of materials or velocities in a process vessel or supply information about transient phenomena in the process. The advantages of such technology, such as high temporal resolution, low cost, no radiation hazard and non-intrusive/non-invasive, have made electrical tomography a promising technology in monitoring and analyzing various industrial flows [1]. Electrical tomography can be further divided into Electrical Capacitance Tomography (ECT) and Electrical Impedance Tomography (EIT), based upon the dielectric properties of the continuous phase in the domain. EIT measurements provide information on the real and imaginary parts of the impedance, as well as the phase angle. However, in the process industries, applications commonly rely on the conductivity difference between two phases to obtain the concentration profiles. Therefore, Electrical Resistance Tomography (ERT) are dominantly employed in imaging conductive flow measurements [2].

Urban wastewater is defined as the mixture of domestic wastewater from kitchens, bathrooms and toilets; other sources of wastewater are industries discharging into sewers and rainwater run-off from roads and other impermeable surfaces such as roofs, pavements and roads draining to sewers [3]. In the UK, the underground sewer system, with a total length of 347,000 km, carries over 11 billion liters of wastewater every day [4]. However, the operational conditions of sewer systems can change over time due to blockages caused by sediment and fats in the untreated wastewater, and structural changes associated with ageing; hence, it is vital to gather sufficient information on the current condition of a sewer system to proactively prevent the service failures. Water companies are now looking for new ways of reducing blockages, and thus the chances of flooding, through means such as modelling to identify hot-spots, monitoring for progressive operational deterioration, and intervening proactively [5]. It has been concluded through statistical analysis that the majority of blockages occur in pipes of diameter 225 mm or less and mostly upstream of a junction [6]. Moreover, the flow velocities can affect the risk of blockages, vice versa. Therefore, flow monitoring at the locations with high likelihood of blockages is essential for continuously inspecting the sewer and identifying the intermittent sewer blockages. Currently, conventional non-tomographic technologies that are only capable of providing flowrate measurements, such as ultrasonic Doppler velocity profilers, electromagnetic meters, Coriolis mass meters, and Venturi meters, are commercially available. However, the wastewater flow behaviour comprises more than one phase due to the complexity of its compositions, which cannot be differentiated by traditional flow meters. Process tomographic scanners, as a result, can be good alternatives by providing real-time cross-sectional images on the phase distribution.

The Environment Agency Monitoring Certification Scheme (MCERTS) regulates the minimum rated operating conditions for fluid conductivity shall be 50 µS cm^−1^ to 1200 µS cm^−1^ [7], which makes ERT a good candidate for playing an important role in monitoring wastewater flow. One of the major concerns in sewers is blockages caused by built-up grease, sanitary products, food scraps, etc. ERT can distinguish between different concentrations by their conductivities; further identification of transitions between homogeneous and heterogeneous flow regime can be implemented by direct/indirect interpretation of measurements. Since obstacles in pipeline systems can disturb the dynamic flow behavior [8], blockages can change the flow within the sensing area. Therefore, by properly characterizing wastewater flow regimes, early detection of sewer blockages can be achieved.

ERT has been heavily applied in multiphase flow studies by the means of estimating the phase fraction through the mixture’s dielectric properties of fluid [9,10] without distorting the flow field, including identifying the flow regimes [11,12,13], visualizing multiphase flow [14,15], mass flow rate measurement [16]. Additionally, ERT sensors can estimate the velocity profiles by cross-correlation between two identical sensors [17,18] The operation principle of ERT is injecting current or applying voltage to electrodes mounted on the boundary of the domain and measuring the voltage or current from the rest of the electrodes. Various ERT hardware systems have been developed as research tools for process engineering studies [10,14,15,19,20,21,22]; a few electrical tomographic systems have also been commercially developed, for instance, MPFM4R&D ECT system from Tech4Imaging [23], SONARtrac ECT flow meter from CiDRA [24], Rocsole ECT pipe sensor from Rocsole [25] and the FLOW-ITOMETER ECT/ERT sensors from itoms [26].

In this paper, an ERT hardware system will be developed, dedicated to wastewater applications. A top-down view of the proposed design will be presented and explained, and each module included in the design will be described in detail. Lab-scaled stationary and dynamic experiments were conducted to verify the accuracy of the measurements. Image reconstructions, including a quantitative image quality analysis, as well as numerical analysis, namely Signal to Noise Ratio (SNR) and cross-correlation, will be presented to evaluate the system performances.

## 2. System Overview

**Figure 1:**
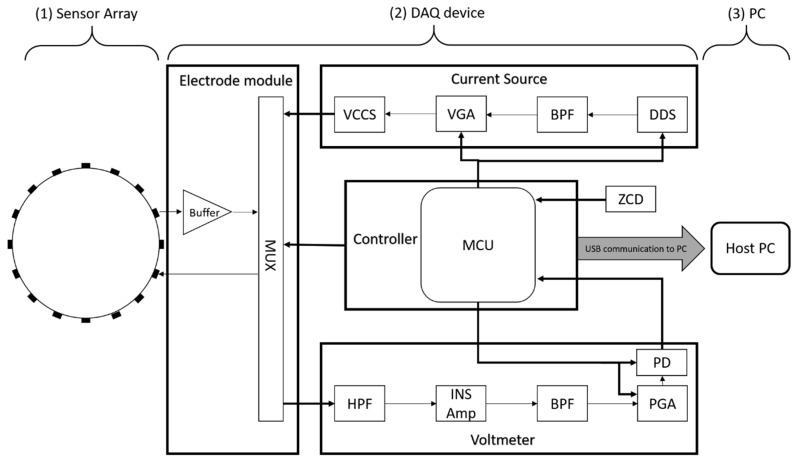
System overview.

An overview of a 16-channel ERT hardware system is presented in Figure 1. The system incorporates: (1) A sensor array equally spaced around the domain periphery; (2) An effective data acquisition system (DAQ) device; (3) A personal computer that interacts with the DAQ, exacting and processing information.

**Figure 2:**
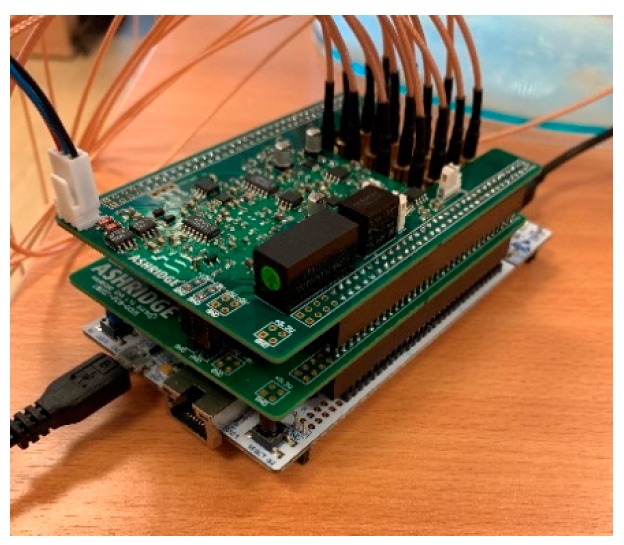
16 channel ERT device.

The proposed DAQ system is a 16-channel serial ERT device, as shown in Figure 2, operating at 50 kHz using the adjacent driving and measuring protocol. It features the user configurable current injection at 50 kHz over the range of 6 mA to 18 mA, as well as the load-adaptive capability. The DAQ system is designed in a modular manner, and may be broken down into four modules:

- An excitation source;
- An electrode switching module;
- A reception module;
- A central control unit functioned by an STM32 microcontroller

These four modules are prototyped on three separate boards, i.e., excitation and reception board (top layer), switching board (middle layer) and microcontroller board (bottom layer). The advantages of this architecture are easy assembly, high mobility, and compactness.

## 3. Design and Methods

### 3.1. Excitation Source

Current, as opposed to voltage, is chosen as an excitation signal to the periphery of the conducting medium on the premise that a current source has a high output impedance, so that the effects of electrode contact impedance will be negligible. The current source presented in this paper is composed of a signal generator, a band-pass filter, a variable gain amplifier and a voltage controlled current converter.

Excitation signals in alternative waveforms have great advantages in reducing the effect of electrode polarization and isolating the direct current offset potential. Sinusoidal waveforms further provide access to full electrical impedance demodulation, and hence, the capability of spectroscopic measurements. Low-cost monolithic devices which can generate accurate waveforms via direct digital synthesis have recently become commercially available. Hence, in the reported design, a Direct Digital Synthesizer (DDS) is selected to produce a sinusoidal signal. A DDS can benefit the system with its fine resolutions at low frequencies (up to 200 kHz), inherent stability, and compactness. However, an unfiltered DDS output signal is biased and rich in spurious content introduced by a Digital to Analogue Converter (DAC). Thus, a fast settling Sixth-order Butterworth Bessel band-pass filter with a passband of 20 kHz centred at 50 kHz follows the DDS.

A variable gain amplifier (VGA) is used to allow for a flexible current excitation within the range of 6 mA to 18 mA. The VGA is essentially a non-inverting amplifier incorporating with a programmable digital potentiometer (DigiPot) and fixed value resistors. The value of DigiPot determines the gain of the VGA and is set via user commands at the beginning of collections referring to a look-up table.

One of the difficulties in designing an ERT system for process applications is the wide range of conductivities, varying from several µS cm^−1^ to a few hundred mS cm^−1^. Hence, a current source that is able to provide a constant current supplying over a wide range of load impedances is a necessity. A dual op-amp voltage controlled current source developed by [27], which is capable of preserving high output impedance over a wide range of operating frequencies, is built around commercially available current feedback amplifiers in our system. The output current is a result of input voltage, $V_{\mathrm{in}}$ and the sensing resistor $R_{\mathrm{sense}}$, following the equation:

(1) $$I_{\mathrm{out}}{=V}_{\mathrm{in}}{/R}_{\mathrm{sense}}$$

### 3.2. Electrode Switching Module

In this system, a serial collection protocol is employed; furthermore, it is critical to have a fast switching mechanism to achieve an overall data acquisition speed. Due to the non-idealities of multiplexers, including the non-zero on-resistance, charge injection, various settling time and retained charge, an appropriately designed switching circuit is essential to not only fulfil fast signal transients but also give the least chance of distorting receiving signals.

A multiplexer with the lowest possible on-resistance is desirable to maintain good linearity. However, the low on-resistance also means a large charge injection, which will result in a voltage glitch occurring at the multiplexer input when switching channels. To compensate for this, the source impedance of a multiplexer should be kept as low as possible. Therefore, a buffer amplifier with a high input impedance and an extremely low output impedance should be placed at each multiplexer input to settle a full-scale step.

A total of four 16:1 multiplexers are exploited, of which two are for the selection source and sink ports and the remaining two for selecting differential voltage ports. Four quadruple low-cost precision Junction gate Field-Effect Transistor (JFET) input operational amplifiers are introduced for buffering the received signal from the electrodes.

### 3.3. Reception Module

#### 3.3.1. Signal Conditioning

Common-mode errors have been considered as being among the most significant sources of measurement errors. As described previously, the proposed design uses a single-end current source topology, the impedance between the grounded side of the current source and the amplifiers in the reception module is the main factor that introduces common-mode signal [28]. To eliminate common-mode errors, two techniques are implemented: (1) an instrumentation (INS) amplifier with a high common-mode rejection ratio (CMRR) at the desired frequency (i.e., 50 kHz) is selected to determine the differential voltage between two voltage signals; (2) fast high pass filters at the inputs of the instrumentation amplifier are used to remove the Direct Current (DC) component of the incoming voltage signals without compromising the acquisition speed.

An Eighth-order Butterworth bandpass filter (BPF) follows the INS amplifier to filter out any distortion that is introduced by the INS amplifier. The BPF is centered at 50 kHz with a 40 kHz passband (−3 dB) and 200 kHz stopband (−40 dB), which results in an approx. 100 µs step response time.

The voltage responses to the current injection can vary over a wide dynamic range from a few millivolts to several volts, depending on the size of the vessel as well as the medium/inclusion in the vessel. To accommodate for diverse applications of the system, another VGA of the same topology as the one employed in the excitation source is incorporated after the BPF, so that the measurements can fall within the acceptable range of the Analog to Digital Converter. This is achieved by a calibration process which happens both at the startup of the system operation and along the whole acquisition process, so that the system can adapt to any unpredictable changes.

#### 3.3.2. Peak Detection

**Figure 3:**
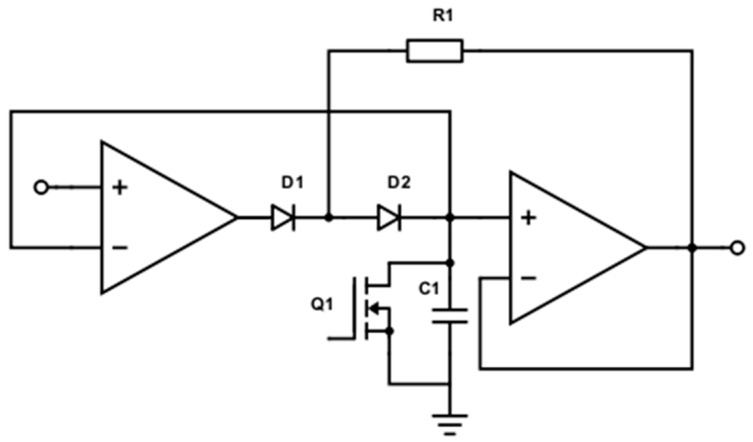
Peak detection circuit.

In an ERT system, only the in-phase component is required for reconstructing the conductivity distribution. An analogue precision peak detection circuit is implemented to obtain the amplitude of the differential voltage, as displayed in Figure 3. The maximum value of the incoming signal is captured by charging up a capacitor, C_1_. Specifically, when the input signal is rising, C_1_ is charged to a new peak level; whereas D_1_ and D_2_ prevent C_1_ to be discharged when the signal is falling. It is also vital to have a reset switch, Q_1_, coupling the capacitor, which is performed by an N-channel enhanced Metal Oxide Semiconductor Field-Effect Transistor, so that the capacitor can be discharged and ready for the next series of signals. The active-high reset signal comes from the microcontroller and will be further discussed in Section 3.4.

The proposed peak detection technique is capable of generating high precision measurements with a high signal to noise ratio. The simplicity, and hence, the cost efficiency of the system, are achieved by replacing the complex phase shift demodulation technique with such peak detection circuit when compared with the conventional ERT device.

#### 3.3.3. Analogue to Digital Converter (ADC)

The general dynamic range of collected signals is from 1:10 to 1:40 [29]; hence, with a minimum 1% accuracy, a 12-bit analogue to digital conversion is necessary. The built-in 12-bit ADC is used to continuously read voltage measurements using Direct Memory Access (DMA) mechanism, which allows for an automatic data transfer from ADC to memory without the usage of the Central Processing Unit (CPU). A total of 35 ADC readings are taken and 5 extrema are eliminated so that each voltage measurement is a result of an average over 30 ADC readings. The averaging technique benefits the system with a better signal to noise ratio by the factor of $\sqrt{N}$ (N is the number of times of averaging).

### 3.4. Central Control Unit

**Figure 4:**
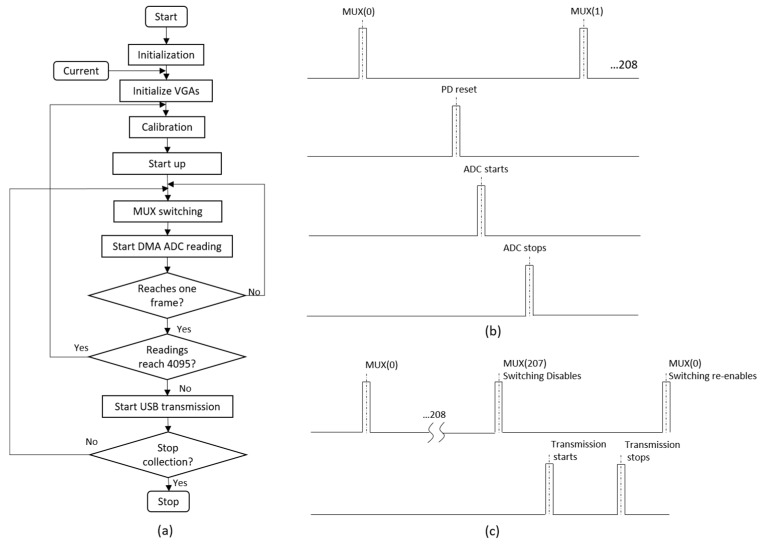
(**a**) Operation procedure of the microcontroller command (**b**) Timing simulation between two switching instances (**c**) Timing simulation between two frames of collection.

An STM32F4 microcontroller handles the commands to programmable integrated circuits (ICs), data exchange between a host PC and the device, and data processing. The sequence of operation follows the flowchart in Figure 4a below. After the system initialization, based on the choice of injection current level from the user command, a VGA gain on the injection side (VGA_i_) is set for current. At the same time, an initial gain is also set for the reception side VGA (VGA_r_), which is normally the smallest value in the look-up table. A calibration process is then performed after a whole frame of data is collected and a corresponding gain for VGA_r_ is calculated. Following that, a startup operation that allows for 5 frames (each frame contains 208 switches) of switching is carried out before the data transmission happens. This startup operation enables the system to settle; hence, a better quality of data will be obtained in the later collection procedure. The authentic measurements then start on the command received at the end of the startup stage. A re-calibration check takes place each time a whole frame of readings is fed into the ADC. If any readings in this frame have reached 4095, then the calibration process will happen again to set a new VGA_r_ gain accordingly.

The sequence of one measurement follows that shown in Figure 4b. The timing is essentially governed by a series of pulses of the stimulation frequency, i.e., 50 kHz, which is generated by the zero crossing detection of the input signal. The multiplexer switches at every 17 cycles of pulses and leaves the system operating at 14 fps. The peak detection reset signal occurs after 9 cycles from the switching instance to give signals sufficient settling time. Subsequently, the ADC conversion happens at cycle 11; the reading and averaging of measurements finishes in 4 cycles.

Communication between the host PC and the ERT device is accomplished via Universal Serial Bus (USB) micro-AB at full speed (12 Mbps). On the host terminal, data could be received and stored either by REALTERM terminal emulator software or MATLAB. It’s worth noting that only after a whole frame of 208 measurements all taken, this one frame of data is transmitted to the host PC at once, as indicated in Figure 4c.

## 4. Experiments

**Figure 5:**
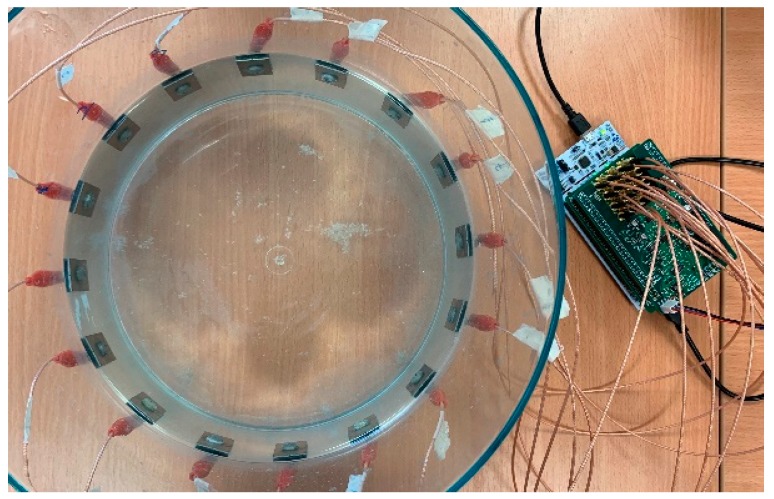
The EIT system for experiments.

To evaluate the performance of the proposed system, multiple experiments were carried out in a cylindrical phantom of 25 cm diameter with 16 electrodes attached evenly on the periphery, as indicated in Figure 5. In all tests, saline is used as a background medium, and is prepared with the solution of NaCl and tap water.

Image reconstructions aiming at recovering conductivity distributions from boundary measurements were performed by field electrical modelling for forward model and inversion algorithm. The electrical conductive field model is governed by the derivation of Maxwell’s equation:

(2) ∇·(σ∇u)=0,

where σ is the conductivity, u is the electric potential distribution, and ∇ is the divergence operator. For the appropriate formulation of the system, the complete electrode model (CEM) is used to constrain the boundary conditions described by:

(3) $$U_{k}{=u+Z}_{k}\sigma \frac{\partial u}{\partial \hat{n}}{\;\mathrm{on}\;e}_{k};\;k=1,2,\ldots ,K,$$

where **u** is the potential field, $Z_{k}$ is the contact impedance of the kth electrode, $\hat{n}$ is the outward unit normal vector and $e_{k}$ denotes the part of boundary that corresponds to the kth electrode. The finite element method (FEM) is a numerical discretizing method commonly used in EIT, and it discretizes the domain of interest into small elements to turn a continuous problem into a discrete problem, and hence solves the forward model. For stable image reconstruction, a linear inverse problem is solved using the Jacobian Matrix J and a Laplacian regularization function leading to matrix R used with regularization parameter $\gamma^{2}$ which is empirically selected. An image of change in electrical conductivity Δσ can be obtained from differential voltage measurements $\Delta {u=u}_{i}-u_{b}$, where $u_{i}$ and $u_{b}$ are the measurements with and without inclusions respectively, following Equation (4):

(4) $$\Delta {\sigma =(J}^{T}{J+\gamma }^{2}{R)}^{-1}J^{T}\Delta u$$

### 4.1. Background Test

**Figure 6:**
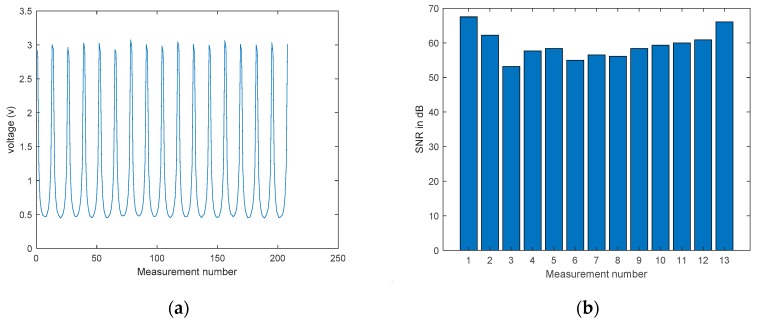
(**a**) Background dataset plot (**b**) SNR plot of 13 measurements in background test.

To help understand the quality of measurements collected from the proposed 16 channel ERT system, a uniform background test is firstly conducted under the lowest possible current injection, 6 mA, and the voltage measurements $u_{b}$ are captured in Figure 6a. Signal-to-Noise Ratio (SNR) evaluates the precision of measurements by indicating the ability to produce the same results under the unchanged conditions [30]; it can be calculated by:

(5) $$\mathrm{SNR}=10\log\frac{{\left[\bar{v}\right]}_{i}}{\mathrm{SD}{\left[v\right]}_{i}}$$

where $\mathrm{SD}{\left[v\right]}_{i}$ is the standard deviation of measurements and ${\left[\bar{v}\right]}_{i}$ is the mean value of total measurements. For one projection from the first excitation using electrodes 1 and 2, the SNR of the corresponding 13 measurements is displayed in the bar chart in Figure 6b. As expected, a higher SNR occurs at adjacent electrode measurements that have a larger trans-impedance, while a lower SNR occurs at opposite-electrode measurements that have a smaller trans-impedance. The overall average SNR over 208 measurements is 59.54 dB, demonstrating the good reliability of the device.

### 4.2. Stationary Tests

#### 4.2.1. Single Sample Tests

Four sets of experimental tests using plastic (Teflon) rods in the sizes of 2 cm (small), 3 cm (medium) and 4 cm (large) diameter, and a plastic ballpoint pen in the size of 5 mm (Xsmall) are reported in this section. In the large and medium sample cases, 6 mA current was injected; whereas 16 mA current was used to stimulate the system in the small and Xsmall sample tests. This is because the signal changes induced by small inclusions are not as significant as those by larger inclusions and are more vulnerable to the background noise. Each object was placed at various locations within the tank, and results are displayed and compared with the real distributions in Table 1, Table 2 and Table 3.

As indicated from Table 1, Table 2, Table 3 and Table 4, the reconstructed images are well defined within the view region, and can reflect the location variations when compared with the real distributions. However, notable image distortion can be observed as objects move further away from the boundary of the domain. This is caused by the inherent ill-posed nature of ERT that it has a high sensitivity to the changes occurring near the boundary. This poses a more significant effect when the targets are rather small and the changes in impedance by objects could be more severely contaminated by noises occurring at the boundary. Hence, even with a stronger excitation, images still struggle to preserve the shape and size of the real images in the small sample test (Table 3) and the Xsmall sample test (Table 4). It is worth noting that even the extra small object, whose size is only 0.04% of the area of the tank, is still detectable by the system. This is benefited from the high SNR of the ERT system and gives the system the confidence of capturing small obstacles and hence potential blockages at an early stage in the field applications.

Quantitative image quality analysis is reported to further compare the reconstructed images with the real distribution. Two evaluation parameters are employed here, i.e., Position Error (PE) and Shape Deformation (SD) from [30], and are plotted against locations. Each image is made up of 200 × 200 pixels and can be represented by a column vector $\hat{x}$; a threshold of one-fourth of the maximum amplitude is applied for the detections of the most visually significant effects:

(6) $${\left[{\hat{x}}_{q}\right]}_{i}=\{\begin{array}{cc} 1, & \;{\left[{\hat{x}}_{q}\right]}_{i}\geq \frac{1}{4}\max(\hat{x})\\ 0, & \;\mathrm{otherwise} \end{array}.$$

1. Position Error

Position error describes the mismatch between the centre of mass of the real distribution $P_{o}$ and the reconstructed image $P_{q}$:

(7) $$\mathrm{PE}=\left|P_{o}-P_{q}\right|$$

**Figure 7:**
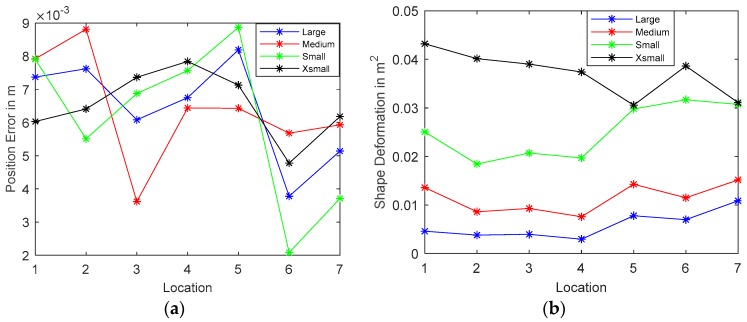
Reconstruction accuracy plots (**a**) Position Error; (**b**) Shape Deformation.

Hence, PE is preferably as small as possible so that it could provide reliable information on the location of blockages in the smart-metering applications. The PE of the four objects are plotted in Figure 7a. Position errors in all tests are managed to be kept below 1 cm, which indicates good tracking of potential blockages.

2. Shape Deformation

Shape deformation measures the fraction of the reconstructed one-fourth amplitude that fails to fit within a circle of the area equivalent to the real image:

(8) $$\mathrm{SD}=\underset{k\notin C}{\sum }{\left[{\hat{x}}_{q}\right]}_{k}/\underset{k}{\sum }{\left[{\hat{x}}_{q}\right]}_{k}$$

where C is a circle centered at $P_{q}$ with an area equal to $A_{q}$. SD should also be low and uniform as large SD may result in incorrect interpretation of inclusions. The SD plots of four samples at various locations are presented in Figure 7b. The ability to preserve the shape and size of objects declines as the objects get smaller, as expected. Moreover, the variation of SD becomes larger as the size of objects increases. The SD performance could be further improved by applying an advanced reconstruction algorithm in offline post-process or applying a size orientated threshold technique.

#### 4.2.2. Multiple Sample Tests

Another set of tests with more than two samples in the tank are also performed in Table 5. The results give a good insight of the distinguishability of the system. When the objects are placed equally close to the boundary, all of them can reveal themselves in the recovered images; however, smaller objects tend to have lower amplitude responses. Difficulties arise when any of the objects were placed in the center of the view region, and objects are effectively merged into one in the reconstructed images (i.e., Location3 and Location6 in Table 5).

### 4.3. Dynamic Tests

In order to study the feasibility of flow monitoring with the framerate offered by the proposed ERT hardware system, dynamic tests were carried out by continuously moving an object in this section. For the purpose of simplification, 2-dimensional flow simulation, which is accomplished using measurements obtained from one electrode plane, is considered.

**Figure 8:**
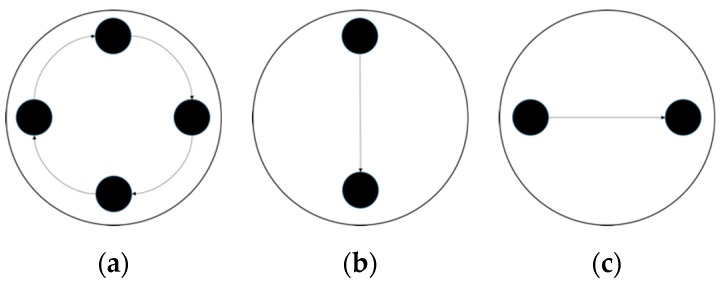
Movement indications of (**a**) circular (**b**) vertical cross (**c**) horizontal cross.

Experiments were conducted using the same phantom setup as single stationary tests in 0 employing the large sample (4 cm diameter plastic rod). Two types of movement are studied: the circular movement where the sample moves along the inner wall of the tank clockwise (Figure 8a); the cross movement where the sample moves across the region along the diameter from one side to another (Figure 8b,c).

#### 4.3.1. Circular Movement

The circular movement provides a good insight into employing ERT into monitoring dynamics within process equipment such as impeller-based mixers, hydro-cyclones and centrifugal separators [31]. Several reconstructed images along the movement path are presented in Table 6. The sample is well defined, even under the continuous movement, and this illustrates that the data capture speed is sufficiently high compared to the rate of movements.

**Figure 9:**
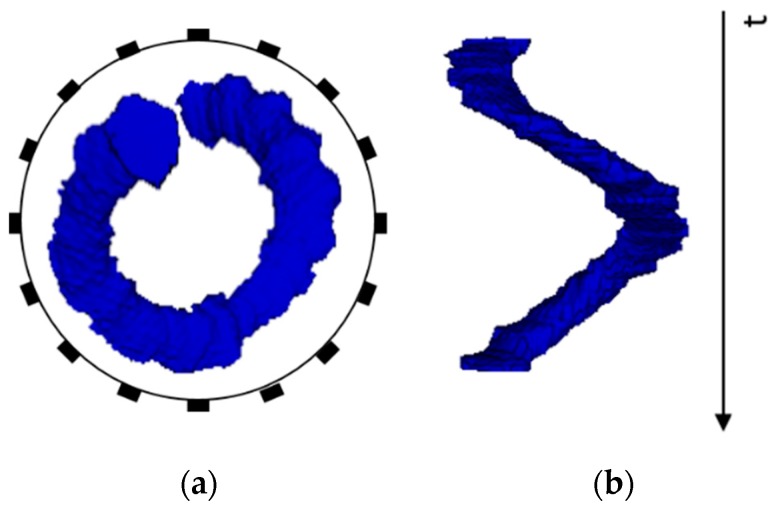
Circular motion 3D volume model (**a**) top view (**b**) helical 3D view with time axis.

To demonstrate the sample movement over the given time margin, a 3D volume model is introduced in Figure 9. In Figure 9a, the top view of the sample motion describes the circular movement within the phantom and a helical 3D volume model in Figure 9b provides the time taken for such movement to complete. The 3D modelling helps not only visualize the movement of targets but obtain volume information given the angular speed derived from the system framerate (will be further discussed in Section 5.1).

#### 4.3.2. Along Diameter Movements

The 2D cross movements can be extended into 3D processes where two electrode planes can be implemented; flow patterns in horizontal slurry transport pipelines [32] can then be investigated. Again, seven critical slices of images of each type of cross movements are displayed in Table 7 and Table 8. The results prove that the movements are successfully monitored.

## 5. Cross-Correlation

**Figure 10:**
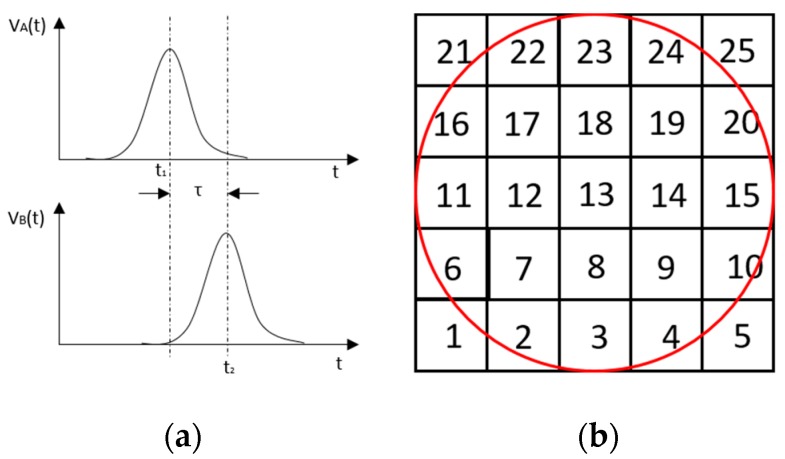
(**a**) examples of signals from pixel A and B; (**b**) indication of pixel units division within the view region.

The resulting ERT images displayed in Section 4.3 provide good insights into the phase distribution as well as transients in two phase flow. Subsequently, a velocity field can be inferred by computing correlations between images of different time instants based on a sequence of reconstructed tomographic images as illustrated in Figure 10a. In a system with one ring of electrodes, the cross-sectional plane of interest is divided into finite elements, which is defined as the pixel correlation method [32], as shown in Figure 10b. Each reconstructed image is composed of these pixels, with each unit having a value indicative of the resistivity of the region it occupies. Then, the profile of individual pixels (i.e., the characteristic vector) can be obtained from the variations of its resistivity measurements over time. By discretely cross-correlating between two targeting pixels’ profiles (Equation (8)), the resulting cross-correlation $R_{\mathrm{AB}}\left(\tau \right)$ peaks at the time representing the delay between signals $V_{A}$ and $V_{B}$, i.e., τ [31].

(9) $$R_{\mathrm{AB}}\left(\tau \right)=\frac{1}{N}\sum_{n=1}^{N}V_{A}(n\Delta {t)V}_{B}(n\Delta t+\tau ),$$

where signal $V_{A}$ and $V_{B}$ are the characteristic vectors of pixels A and B that the cross-correlation is applied to; N is the length of vector, and Δt is the time-step.

### 5.1. Circular Movement

**Figure 11:**
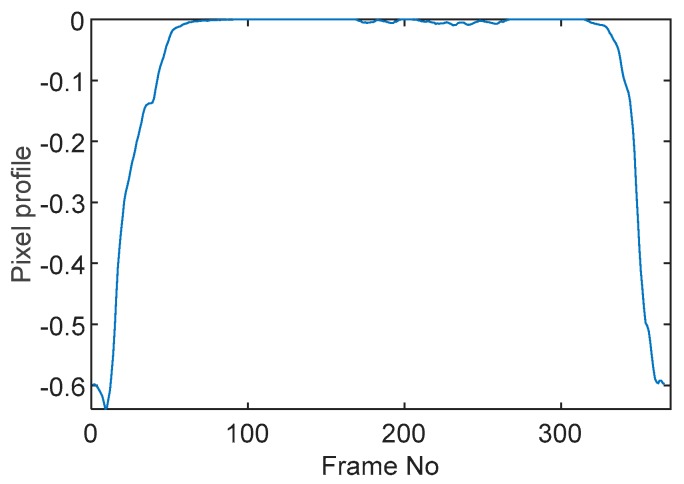
Characteristic value vector plot of pixel 23.

The circular movement starts from pixel 23 and completes one lap in 366 frames. Therefore, pixel 23 is set as a reference whose characteristic vector against frame number is plotted in Figure 11. The pixel profile plots provide information of the time instances when the sample enters and leaves the corresponding pixels. In the example of pixel 23, as described in Figure 11, the sample was placed at pixel 23 before the beginning of capturing and started moving out of the pixel after frame 10 until frame 57 when the object was completely out of this pixel; the object appeared back in pixel 23 from frame 324. The circular movement finishes one lap within 366 frames, which can be calculated as 26.14 s given that the data capture speed is 14 fps. This is comparable to the actual time spent on completing one lap, which is measured by a timer, i.e., 26.38 s.

Six pixels along the circular path are chosen to correlate with the reference pixel and results are displayed in the time-shifting manner in Table 9. Transit time can then be referred to the frame number of the peak in each cross-correlation plot. The time-step of adjacent frames, which is the reverse of the framerate of DAQ device is given, and the speed calculation is hence attainable.

The frame numbers of the peaks in the cross-correlation plots in Table 9 are 72, 107, 147, 181, 225 and 269 in the displayed order. The increase in the peak frame numbers in the cross-correlation plots indicates the time shifting.

The angular speed can be performed by:

(10) $$\omega =\frac{\theta }{{\tau \times v}_{\mathrm{DAQ}}},$$

where θ is the angular distance between two pixels in radius, τ is the transit time in frames and $v_{\mathrm{DAQ}}$ is the data acquisition speed which is 0.072 s per frame. For simplification, a quarter of one lap, which is from pixel 23 to pixel 15, is considered. The cross-correlated transit time, τ, is 107 frames; hence, the angular speed can be calculated as 0.207 rad/s. The accuracy of the speed calculation can be analyzed in comparison with the measured angular speed. The measured time taken for the object to travel over the angular distance of π/2 is 7.81 s, which yields an average angular speed of 0.201 rad/s. Therefore, the relative error of using the cross-correlation method is 2.98% when compared with the measured speed; this is acceptable and proves that the strategy of using cross-correlation to obtain the speed measurements is feasible. The error is partially contributed by the imaging algorithm as the cross-correlation is essentially accomplished from a sequence of reconstructed images and the image quality can play an important role in the success of cross-correlation results. Also, the random error of timing the instance when the object arrives at 90-degree location can result in the difference between the measured time and the cross-correlated time, thereby giving the corresponding speeds.

### 5.2. Along Diameter Movements

**Figure 12:**
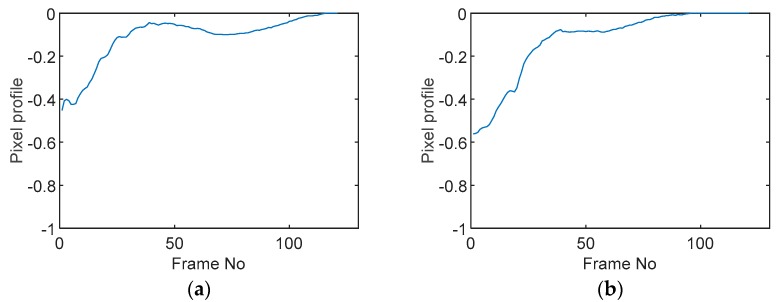
Characteristic value vector plot of (**a**) pixel 23; (**b**) pixel 11.

Tests were also conducted by moving the object across the domain in vertical and horizontal directions, as mentioned previously. In these tests, it started at pixel 23 in the vertical direction and arrived at the opposite side of the tank, i.e., pixel 3, within 121 frames; started at pixel 11 in the horizontal direction and arrived at pixel 15 in 121 frames of time. Thus, pixel 23 and 11 are set as reference and their characteristic vectors are plotted in Figure 12a,b respectively.

Three pixels along the moving path in each case are chosen to cross-correlate against the reference pixels. Similar to the circular movement, the cross-correlation plots peak at the frame numbers that represent the travel time spent on moving from the reference pixels to the corresponding pixels. Results are shown in Table 10 and Table 11.

The frame numbers of the peaks of pixel 18, 13 and 8 cross-correlating to the reference pixel are 9, 51 and 82 respectively in the vertical movements; the frame numbers of the peaks in the cross-correlation plots of pixel 12, 13 and pixel 14 against pixel 11 are 41, 73 and 97.

The speed at which the sample travels along the path can be derived by the same principal as Equation (9) but with the metric distance between two targeting pixels instead. To illustrate, the cross-correlated average speed is calculated at which the sample travels from the reference pixel to the centre (pixel 13) in both the vertical and horizontal directions. Again, the accuracy analysis is shown in Table 12.

The relative errors of along diameter movements are slightly higher than those of circular movements, yet can still provide the confidence of employing cross-correlation to measure speeds. One of the reasons for to the larger relative errors is the image quality. As discussed, the image quality is one of the most vital factors that determine the accuracy of the cross-correlated speeds. Circular movements occur near the periphery of the tank whereas the cross movements involve the object travelling across the whole domain. The inherent difficulties of recovering the objects placed away from the center lower the accuracies.

## 6. Conclusions

Monitoring water supplies is economically and environmentally important, however, it is also costly. Blockages of pipes or flow restrictions are common; whether it is wet wipes in sewage networks, or gravel and silt clogging in pipes, it is important to know where these blockages occur (or are likely to occur), to facilitate maintenance and minimize disruptions to the networks. This work reports an ERT system demonstrating the use of sensors to image pipes with different flow constituents including solids and liquids. The impact of this work is an embedded sensor monitoring the quality and rate of flow in water and sewage networks, feeding into water quality and availability improvements.

The device incorporates a serial measurement structure with a common mode error cancellation method to allow for a cost-effective but fast data collection design. Furthermore, it features the flexibility of injecting current from 6 mA to 18 mA whilst being able to respond to the wide dynamic load range. A data collection rate of 14 fps, as well as an overall mean SNR of more than 59.54 dB, has been achieved. The system performances in both the static and dynamic range were studied in lab-scale experiments. With the smallest sample size reported being 0.04% of the phantom, the system managed to locate inclusions with a position error of less than 1 cm. Also, successful motion tracking was achieved by clear visualization of the movements, as well as numerical speed calculation with less than 4% relative errors.

## Figures and Tables

**Table 1:** Large sample tests.

Location1	Location2	Location3	Location4	Location5	Location6	Location7
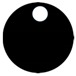	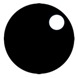	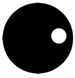	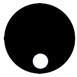	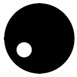	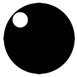	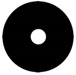
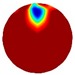	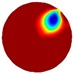	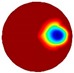	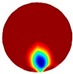	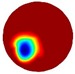	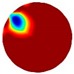	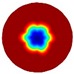
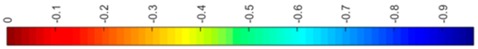						

**Table 2:** Medium sample tests.

Location1	Location2	Location3	Location4	Location5	Location6	Location7
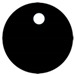	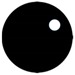	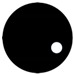	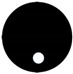	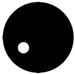	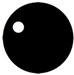	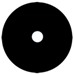
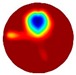	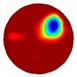	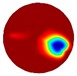	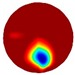	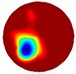	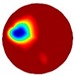	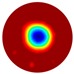
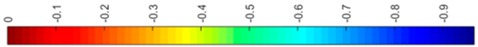						

**Table 3:** Small sample tests.

Location1	Location2	Location3	Location4	Location5	Location6	Location7
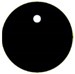	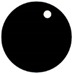	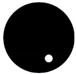	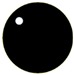	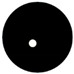	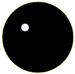	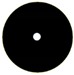
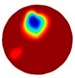	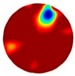	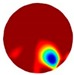	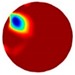	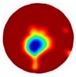	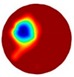	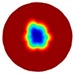
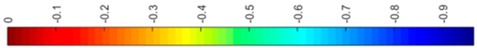						

**Table 4:** Extra small (Xsmall) sample tests.

Location1	Location2	Location3	Location4	Location5	Location6	Location7
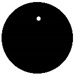	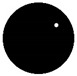	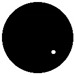	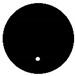	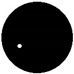	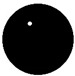	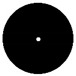
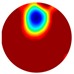	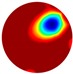	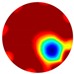	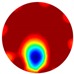	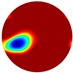	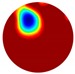	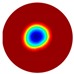
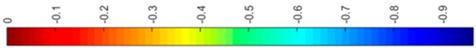						

**Table 5:** Multiple sample tests.

Location1	Location2	Location3	Location4	Location5	Location6	Location7
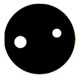	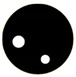	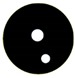	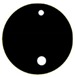	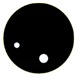	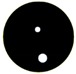	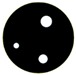
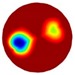	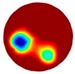	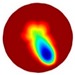	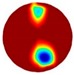	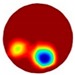	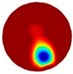	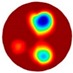
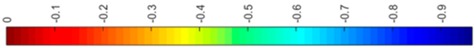						

**Table 6:** Large sample circular movement test reconstructed images.

Location1	Location2	Location3	Location4	Location5	Location6	Location7
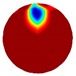	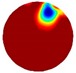	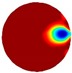	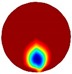	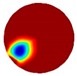	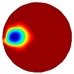	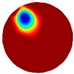
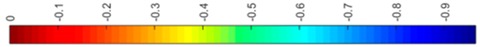						

**Table 7:** Large sample vertical cross movement test reconstructed images.

Location1	Location2	Location3	Location4	Location5	Location6	Location7
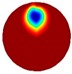	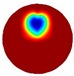	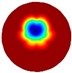	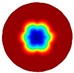	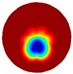	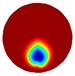	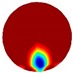
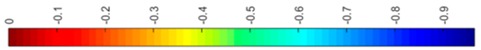						

**Table 8:** Large sample horizontal cross movement test reconstructed images.

**Location1**	**Location2**	**Location3**	**Location4**	**Location5**	**Location6**	**Location7**
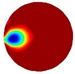	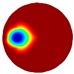	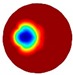	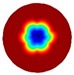	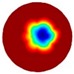	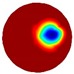	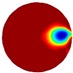
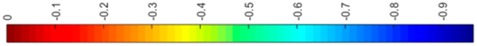						

**Table 9:** Pixel characteristic vector plots and cross-correlation plots of circular movement.

	**Pixel 20**	**Pixel 15**	**Pixel 9**
**Pixel profile plot**	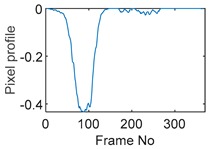	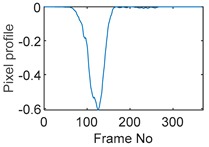	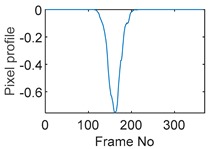
**Cross-correlation**	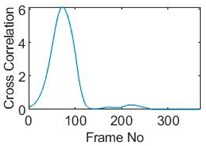	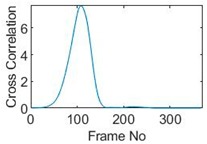	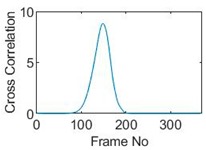
	**Pixel 3**	**Pixel 7**	**Pixel 11**
**Pixel profile plot**	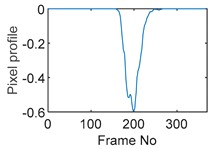	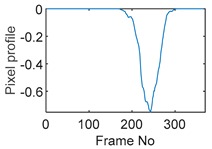	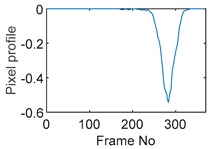
**Cross-correlation**	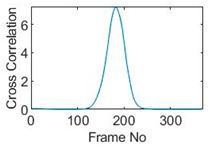	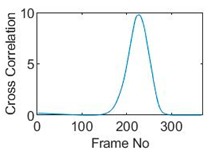	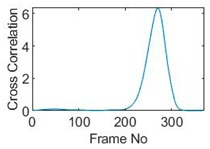

**Table 10:** Pixel characteristic vector plots and cross-correlation plots of vertical cross movement.

	Pixel 18	Pixel 13	Pixel 8
**Pixel profile plot**	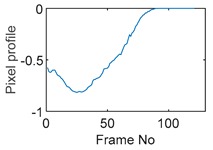	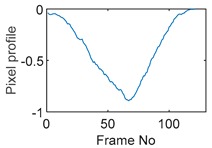	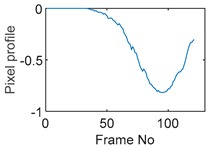
**Cross-correlation**	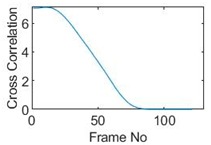	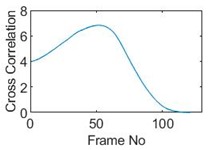	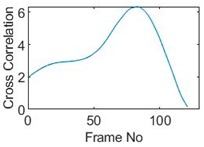

**Table 11:** Pixel characteristic vector plots and cross-correlation plots of horizontal cross movement.

	Pixel 12	Pixel 13	Pixel 14
**Pixel profile plot**	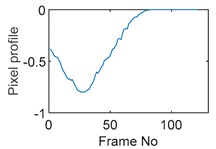	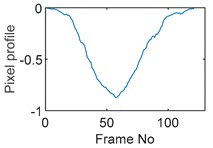	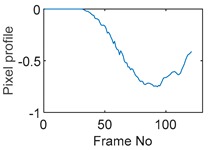
**Cross-correlation**	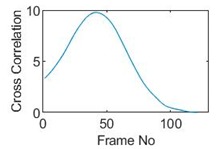	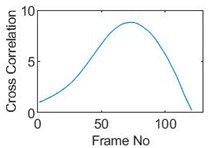	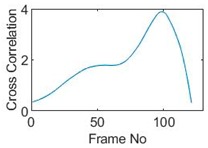

**Table 12:** Speed results of cross movements.

	Measured Speed (m/s)	Cross-Correlated Speed (m/s)	Relative Error (%)
Vertical	0.0265	0.0274	3.39
Horizontal	0.0186	0.0192	3.22
